# Application of diagnostic network optimization in Kenya and Nepal to design integrated, sustainable and efficient bacteriology and antimicrobial resistance surveillance networks

**DOI:** 10.1371/journal.pgph.0002247

**Published:** 2023-12-06

**Authors:** Marie Brunetti, Amit Singh, Sheilla Chebore, Deepak Gyenwali, Nada Malou, Cecilia Ferreyra, Tulsi Ram Gompo, Sharmila Chapagain, Susan Githii, Evelyn Wesangula, Heidi Albert

**Affiliations:** 1 FIND, Geneva, Switzerland; 2 FIND, Bangalore, India; 3 FIND, Nairobi, Kenya; 4 FIND, Kathmandu, Nepal; 5 Central Veterinary Laboratory, Kathmandu, Nepal; 6 National Antimicrobial Stewardship Interagency Committee, Nairobi, Kenya; 7 FIND, Cape Town, South Africa; Aga Khan University, PAKISTAN

## Abstract

Antimicrobial resistance (AMR) is a major global public health concern, particularly in low- and middle-income countries, which experience the highest burden of AMR. Critical to combatting AMR is ensuring there are effective, accessible diagnostic networks in place to diagnose, monitor and prevent AMR, but many low- and middle-income countries lack such networks. Consequently, there is substantial need for approaches that can inform the design of efficient AMR laboratory networks and sample referral systems in lower-resource countries. Diagnostic network optimization (DNO) is a geospatial network analytics approach to plan diagnostic networks and ensure greatest access to and coverage of services, while maximizing the overall efficiency of the system. In this intervention, DNO was applied to strengthen bacteriology and AMR surveillance network design in Kenya and Nepal for human and animal health, by informing linkages between health facilities and bacteriology testing services and sample referral routes between farms, health facilities and laboratories. Data collected from the target settings in each country were entered into the open-access DNO tool OptiDx, to generate baseline scenarios, which depicted the current state of AMR laboratory networks and sample referral systems in the countries. Subsequently, baselines were adjusted to evaluate changing factors such as samples flows, transport frequency, transport costs, and service distances. Country stakeholders then compared resulting future scenarios to identify the most feasible solution for their context. The DNO analyses enabled a wealth of insights that will facilitate strengthening of AMR laboratory and surveillance networks in both countries. Overall, the project highlights the benefits of using a data-driven approach for designing efficient diagnostic networks, to ensure better health resource allocation while maximizing the impact and equity of health interventions. Given the critical need to strengthen AMR laboratory and surveillance capacity, DNO should be considered an integral part of diagnostic strategic planning in the future.

## Introduction

Antimicrobial resistance (AMR) is a major global public health concern and growing rates of drug resistance threaten our ability to treat even common infections [1]. Drug-resistant infections impose huge costs on individuals and society as they spread between the environment, animals and humans [1, 2]. A recent landmark study on the global burden of AMR found that drug-resistant bacteria directly caused 1.27 million deaths and were associated with a further 4.95 million deaths in 2019 [3]. It has been estimated that up to 10 million people each year will die as a result of AMR by 2050 unless urgent action is taken [2].

The burden of AMR is particularly high in low- and middle-income countries, as a result of complex, interplaying factors. Low- and middle-income countries experience a high burden of infectious diseases, but may lack adequate health systems, laboratory capacity, and the resources required for surveillance of such diseases and diagnostic testing to inform appropriate antimicrobial use [3–8]. As a result, antibiotic misuse is high in low- and middle-income countries, where most antibiotic prescriptions are made by doctors and other medical professionals based on empirical diagnosis, without using a diagnostic tool to confirm the underlying infectious agent [9, 10]. Consequently, AMR is further driven by the overuse of antibiotics, which encourages the spread of drug-resistant bacteria. Confounding factors, such as poverty, overcrowding and improper handling of contaminated waste in the environment can further contribute to AMR [8]. Tackling AMR for human and animal health in low- and middle-income countries is therefore part of a wider challenge related to health and diagnostic system strengthening.

There are numerous tests to identify infectious agents such as bacteria and fungi and test their susceptibility to different antimicrobial agents. Common methods include Gram staining, culture, biochemical testing and manual antimicrobial susceptibility testing [11]. More modern methods include testing on automated platforms such as the VITEK system, and genomic sequencing [12]. However, in many countries these complex devices may only be available at higher tiers of the healthcare network (e.g. at a reference or the central laboratory), or may not be available at all in lower-resource settings [13]. This is a particular issue in severe and life-threatening conditions like sepsis, where the availability of diagnostics is critical, as the risk of death for the patient increases by an estimated 7.6% with every hour that passes before treatment begins [14].

As a result, laboratory networks and sample referral systems between national, regional, and peripheral facilities are critical for ensuring accessible and timely diagnostic testing. Efficient and accessible diagnostic laboratories and sample referral systems at the lower levels of the health system are particularly important for patient care, as healthcare practitioners require timely results from the identification of pathogen and antimicrobial susceptibility testing to effectively treat patients with the right antimicrobial agent. Such networks are also vital for AMR surveillance, for example, to monitor community outbreaks of multidrug-resistant bacteria and inform disease control activities and treatment prescribing guidelines at a local level. AMR surveillance is typically conducted using bacterial isolates sent via sample referral systems to higher levels of the network. However, many low- and middle-income countries do not have organized sample referral systems for bacteriology testing and AMR surveillance, and patients may be referred to higher levels of the health system to access these services. When they exist, sample referral systems often are fragmented and not integrated across diseases. In some cases, there may be no access to suitable laboratories for testing.

Strengthening the capacity for bacteriology testing and AMR surveillance in low- and middle-income countries therefore requires key changes to how testing is handled at all levels of the health system and the implementation of a One Health approach that recognizes the interconnection between people, animals, plants, and their shared environment [15]. Firstly, there is a critical need for rapid diagnostic solutions in primary care, simplified blood culture methods and antimicrobial susceptibility testing at higher levels of the network, as well as digital solutions to improve result interpretation and support clinical decisions. Secondly, there is a need to improve access to and optimize the use of existing tests in healthcare systems for both animal and human health. In particular, there is a need for systematic approaches to inform the placement and use of diagnostic instruments, human resources and sample referral linkages.

Diagnostic network optimization (DNO) is a geospatial network analytics approach to plan diagnostic networks consistent with national health goals and strategies [13, 16]. DNO enables analysis of the current diagnostic network and the development of evidence-based recommendations around the optimal type, number and location of diagnostics and an associated sample referral network that maximize access to and the overall efficiency of the health system [13]. DNO is being increasingly used in the public health sector to inform diagnostic instrument placement, optimize sample transportation and referral mechanisms, facilitate geographical prioritization and efficient integration of testing to meet the priority needs of a disease programme [13, 17–21]. To date, DNO has largely been used to optimize diagnostic networks for patient management.

This intervention applied DNO to the design of surveillance networks in two low- and middle-income countries–Kenya and Nepal–looking to strengthen and optimize their diagnostic networks for AMR. Specifically, DNO modelling was used to inform the optimal design of AMR laboratory and sample referral networks in both countries, to improve the coverage and efficiency of AMR surveillance. Here, we outline the process used to conduct the DNO analysis and key insights generated from this novel application of DNO to diagnostic networks for AMR.

## Methods

In this intervention, DNO analyses were conducted in Kenya and Nepal between July 2021 and March 2022 to inform the design of bacteriology and AMR surveillance networks for human health in Kenya and for animal health in Nepal. The intervention was conducted in collaboration with the respective Ministries of human and animal health and with the Fleming Fund country grant offices. DNO was conducted in line with established methodology [13] and according to each country’s context and objectives. The intervention aimed to optimize linkages between health facilities and bacteriology testing services at different levels of the health system and assist with the planning of routes for referring samples and isolates between farms, health facilities and laboratories.

### Ethics statement

As this intervention did not include human participants, ethics approval was not obtained.

### Inclusivity in global research

Additional information regarding the ethical, cultural, and scientific considerations specific to inclusivity in global research is included in the S1 Checklist.

### Setting

Kenya’s healthcare system is structured across six tiers, starting with community and primary care, up to primary, secondary and tertiary hospitals [22]. Primary and secondary hospitals (Levels 4 and 5) offer a wide range of services and receive high volumes of patients referred from lower-level facilities. Kenya is enrolled in the World Health Organization’s Global Antimicrobial Resistance and Use Surveillance System (GLASS) and has five AMR surveillance sites participating in the national surveillance system [23]. The existing national AMR surveillance strategy focuses on monitoring trends in bacterial resistance to antibiotics, through a network composed of 14 AMR human health laboratories, including the National Microbiology Reference Laboratory (NMRL), located in 13 counties, with plans for expansion to all 47 counties [24]. Testing is typically done on-site through walk-in or referred patients, as bacteriology sample referrals for patient management are not in place and only a few isolate referrals exist. Although Kenya has established diagnostic networks and strategic plans for the management of tuberculosis (TB) and HIV [25, 26], existing sample referral systems for TB and HIV are complex and fragmented, with varied strategies and implementation approaches across donors and implementing partners.

In Nepal, passive or clinical AMR surveillance for animal pathogens started as early as 2011 [27]. The programme has since expanded to seven animal health laboratories located in six provinces. Currently, Nepal’s Central Veterinary Laboratory (CVL) is also leading active AMR surveillance in market-age chickens including the veterinary laboratories located in the provinces. AMR surveillance is monitored by CVL through confirmatory testing, antimicrobial susceptibility testing and external quality assurance of isolates from animals across the surveillance network. Nepal is also enrolled in GLASS-One Health, a World Health Organization integrated multi-sectoral surveillance system based on the extended-spectrum beta-lactamase E. coli Tricycle project to detect ESBL-producing E. coli across humans, poultry, water bodies. CVL has coordinated with the animal component for the WHO ESBL Tricycle project for detecting ESBL *E*. *coli* from healthy chicken samples [23].

However, Nepal currently lacks an organized referral system for bacteriology testing and AMR surveillance. As specimens are not always referred, farmers directly submit specimens to the nearest laboratory using their own transport system and testing is done on-site. Referral of bacterial isolates for confirmatory testing at the Central Veterinary Laboratory is also not commonly practiced. However, the start of active AMR surveillance in the animal health sector could support in strengthening the One Health approach in AMR surveillance and response [28], which this project set out to assist with planning and implementing.

### Objectives and expected outcomes

The main objectives of the DNO intervention were to:

1. estimate projected future demand for bacteriology and antimicrobial susceptibility testing and determine the optimal capacity and placement of diagnostics to meet future needs in a cost-efficient manner;
2. use established country diagnostic networks to model future expansion of referring facilities within the network; and
3. develop optimal sample transport mechanisms for isolates referred from sentinel sites to national reference laboratories to maximize surveillance coverage.

### DNO tool: OptiDx

The DNO analysis was conducted using OptiDx, a web-based, open-access network analytic tool, designed for use in low- and middle-income countries [29]. OptiDx models the impact of simultaneously changing multiple parameters of network design and performance so multiple scenarios can be compared and evaluated. The tool enables users to input data related to testing sites, demand, tests, costs, and policies that define sample referral linkages and transport, to create a digital model of a country’s current diagnostic network at baseline [30]. Users can then adjust inputs and apply constraints to create alternative network designs, known as scenarios. OptiDx produces filterable tables, maps, and graphs that help depict and answer questions about the location and quantity of testing sites, the location and volume of testing demand, test types, costs, and sample referral routes, modes and volumes. The tool also enables potential network configurations to be compared in terms of access, device utilization and costs, to help identify the ‘best-fit’ solution for the setting. OptiDx is currently in use across low- and middle-income countries to support DNO analysis for various diseases including COVID-19, HIV, TB and human papillomavirus.

### DNO process

A DNO analysis typically comprises five key steps [13]. An overview of the process followed in Kenya and Nepal for each key step is outlined in Table 1.

**Table 1:** Key components of DNO process undertaken in Kenya and Nepal.

Step	Details
**Define scope**	• Define scope of analysis in Kenya and Nepal in collaboration with key stakeholders
**Collate and prepare data**	• Identify data sources • Develop template for data collection • Collate and clean data
**Conduct baseline analysis of diagnostic network for animal and human health to determine the existing routing, current demand, device placement and frequency of isolate referrals**	• In Kenya, conduct baseline analysis of diagnostic networks for human health, device footprint, testing capacity and referral linkages • In Nepal, conduct baseline analysis of diagnostic networks for animal health, existing device footprint, testing capacity and referral linkages • Map available country data on testing and overlay with the existing network to understand gaps and opportunities for improved coverage and efficiency of the network, and referral integration
**Run customized optimization scenarios based on current testing and plans for active surveillance and compare network outputs**	• Estimate the growth in testing demand and assess scenarios to meet the projected demand using the available device footprint and potential procurement of additional devices • Project future demand for bacteriology and confirmatory testing and recommend optimal referral design, including options for integration into existing networks for other samples, transport modes, costs and resource estimates • Compare expected impact of various network designs and implementation strategies
**Select outputs for implementation and to support decision-making**	• Establish national datasets and dynamic network models for human and animal health that can be interrogated for future scenarios for bacteriology and antimicrobial susceptibility testing • Use model outputs to inform national action plans and implementation planning • Support the development of tools/documents on outputs of the DNO exercise to inform cost and design of sample referral systems, including integrated referral systems

#### 1. Scope of analysis

In both countries, DNO analyses were initiated in 2021, which was considered the “baseline” for the AMR and bacteriology networks.

**Fig 1:**
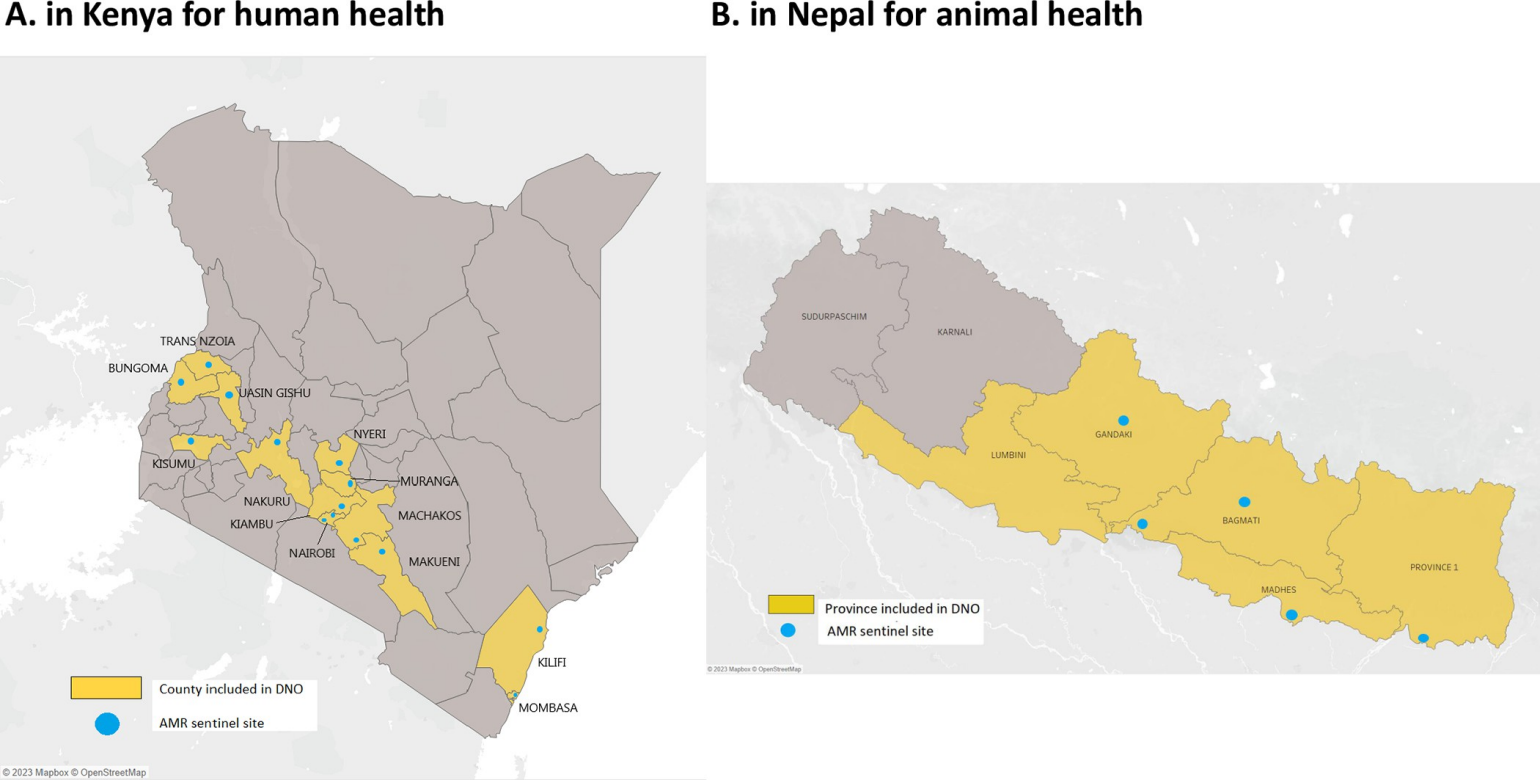
Geographical scope of DNO analysis.

*Kenya*. In Kenya, the scope of the DNO analysis was defined in collaboration with the National Antimicrobial Stewardship Interagency Committee (NASIC) and key stakeholders to ensure alignment with the priority needs of the country. Kenya’s Ministry of Health requested that the analysis focus on AMR networks for human health only. Stakeholders agreed that the analysis would focus on including AMR within the integrated sample referral system, which is currently being implemented in the country. As such, the DNO analysis focused on 13 counties with an AMR sentinel surveillance site (shown in Fig 1A) and covered testing for bacteriology/AMR, TB and HIV. AMR sentinel surveillance sites are usually a hospital laboratory, which can collect specimens, conduct testing and transport isolates to the NMRL and report surveillance data based on antimicrobial susceptibility testing conducted. Specific tests included in the analysis were: Gram stain, culture, bacterial identification, antimicrobial susceptibility testing, and quality assurance/confirmatory testing for bacteriology/AMR at NMRL; GeneXpert MTB/RIF and culture/drug susceptibility testing for TB; and early infant diagnosis for HIV. Previous DNO work conducted with Kenya’s National TB Programme was also used to inform the current project [31].

*Nepal*. In Nepal, discussions were initiated with government authorities and stakeholders to introduce DNO and OptiDx and understand priorities for AMR surveillance. The most important need identified was to strengthen and institutionalize the referral linkages between sentinel sites and reference laboratories to improve AMR surveillance in animal health. Available data on AMR testing and surveillance in Nepal were reviewed to inform the development of a quantified scope of work for the DNO analysis. It was agreed that the analysis would include culture, bacterial identification, and antimicrobial susceptibility testing on samples from poultry species across the geographical scope shown in Fig 1B. This analysis included five out of the seven veterinary laboratories, as the two remaining veterinary sentinel sites did not share any data, and 331 farms and slaughterhouses were mapped to those testing sites.

Base map and data from OpenStreetMap and OpenStreetMap Foundation.

#### 2. Collation and preparation of data for analysis

In Kenya and Nepal, data templates were developed to collect information and key metrics to help evaluate existing bacteriology sample referral and AMR surveillance networks, as of 2021. Table 2 outlines the key data that were collected for Kenya and Nepal. Multiple data sources were used to map the 2021 baseline network, including literature review documentation, programmatic reports and laboratory datasets.

**Table 2:** Key data collected to inform DNO analyses in Kenya and Nepal.

KENYA	NEPAL
Human health	Animal health
14 sites in 13 counties	5 sites in 4 provinces
• Routes, frequency and transport modes used for TB/HIV sample referrals • Locations of microbiology laboratories and TB/HIV testing sites (geocoordinates) • Human resource capacity in microbiology laboratories (list and number of staff engaged in diagnostic and antimicrobial susceptibility testing) • Bacteriology and antimicrobial susceptibility tests performed (culture, identification, antimicrobial susceptibility testing, etc.) • Existing testing capacity at microbiology laboratories (number of devices, tests performed on each device, etc.) • Transport costs • Test costs	• Locations of farms (no geocoordinates, information at ward level) • Locations of veterinary laboratories (geocoordinates) • Human resource capacity in veterinary laboratories (list and number of staff engaged in diagnostic and antimicrobial susceptibility testing) • Bacteriology and antimicrobial susceptibility tests performed (culture, identification, antimicrobial susceptibility testing, etc.) • Existing testing capacity at veterinary laboratories (number of devices, tests performed on each device, etc.) • Transport costs • Test costs

AMR, antimicrobial resistance; TB, tuberculosis.

In Nepal, there were substantial challenges with data availability and quality, notably the non-availability of the geocoordinates for farms. Fictive locations of farms were created using the geographic information system QGIS. The geographic information system (GIS) file of ward distribution was imported into QGIS software to get ward surface areas and coordinates, and a number of farms were assigned to each ward using the random points inside polygon function. Farms were then placed at the centroid of the ward in urban municipalities, and randomly distributed within the ward in rural municipalities. Those farm locations were then converted to geocoordinates and imported into OptiDx for analysis.

#### 3. Building of baseline model

The data collected in step 2 were entered into OptiDx to map current bacteriology diagnostic and AMR surveillance networks in Kenya and Nepal (as of 2021). The baseline analyses gave a geographical representation of the diagnostic networks and helped determine the existing routing, current testing demand, device placement and frequency of isolate referrals for human health in Kenya and for animal health in Nepal. Findings from the baseline assessment were presented to the respective partners in Kenya and Nepal for feedback to help inform the design of future optimized scenarios and future analyses as the networks evolve.

#### 4. Development of customized optimization scenarios

Based on the baseline analyses and feedback collected from partners, optimization scenarios were created using OptiDx. In the optimized scenarios, current bacteriology and AMR networks were overlayed with existing TB/HIV referral systems to identify gaps and opportunities for specimen referrals and integration across diseases in Kenya. Several optimized referral networks were modelled, for samples and isolates referred between farms, veterinary sentinel sites and the Central Veterinary Laboratory in Nepal, and between health facilities, human health sentinel sites and the National Microbiology Reference Laboratory in Kenya, and then compared based on costs associated with the deployment of the different sample referral system designs.

#### 5. Selection and implementation of DNO outputs

Final DNO outputs were presented to the respective Ministries of Health and stakeholders in Kenya and Nepal. In Kenya, outputs were evaluated to determine the most efficient sample referrals through integrating needs for bacteriology/AMR sample referral systems with existing systems established for other diseases. DNO allowed stakeholders in Kenya and Nepal to adjust inputs and constraints and weigh up different elements of each scenario to identify the preferred solution for their context. In Kenya, the recommended county referral systems will be used for the establishment of sample referrals between selected sites and AMR laboratories, and will support the Referral System Technical Working Group in negotiations with contracting courier services to provide sample referral services for all specimens. In Nepal, the findings will be used to implement a pilot of recommended scenarios for the sample referral system across the entire animal health surveillance network before wider scale-up. Detailed transport costings were created to support the interventions in both countries.

## Results

Key insights from baseline and optimal network designs for AMR in Kenya and Nepal are described here.

### Kenya

#### Baseline

Between January and December 2021, a total of 100,294 bacteriology and antimicrobial susceptibility tests in the 14 AMR human health laboratories were conducted in Kenya. Gram stain and culture testing accounted for 71% of the total demand for bacteriology testing. Around 80% of the total AMR testing volume in Kenya was undertaken by just three out of the 14 laboratories (Moi Teaching Hospital in Uasin Gishu, Kenyatta National Hospital in Nairobi and Machakos Hospital in Machakos). The overall average device utilization was low (6%) and varied from 1% to 24% across sites. Across devices, CO2 incubators had the highest utilization rates (17.5%). MALDI-TOF, which is present only at NMRL and one surveillance site, had low utilization (1.2%), as did the automated blood culture devices that are available in most surveillance sites (1.5%). Key reasons for low device utilization and demand were unserviced equipment, a lack of staff training, and high facility workload.

Across the 13 AMR surveillance sites in the network in 2021, six of them sent isolates to the NMRL for quality assurance and confirmatory testing during Q1 to Q3 2021 via the courier drop-off sites (G4S, shown in orange lanes in S1 Fig). The overall volume of isolates sent at baseline was low (64 isolates) but is already increasing as AMR surveillance expands and isolate referrals and shipments to NMRL become a common practice across all sentinel sites.

#### Future state scenarios & recommendations

In Kenya, 89 Level 4 and 5 hospitals across 13 counties were identified as health facility candidates to be linked with the AMR sentinel sites for sample transportation for patient management. Most hospitals are already connected to the AMR sites, which are county or sub-county referral hospitals, through samples referred for rapid TB or HIV molecular testing conducted at AMR sites, or through samples that are pooled at AMR sites and further transported to a testing site. Additionally, some AMR sites are already connected to the NMRL through sample referrals for TB culture and drug susceptibility testing (depicted in S2 Fig), allowing for bacteriology sample and isolate referrals to be integrated into the pre-existing services supported by TB/HIV programmes and partners.

**Fig 2:**
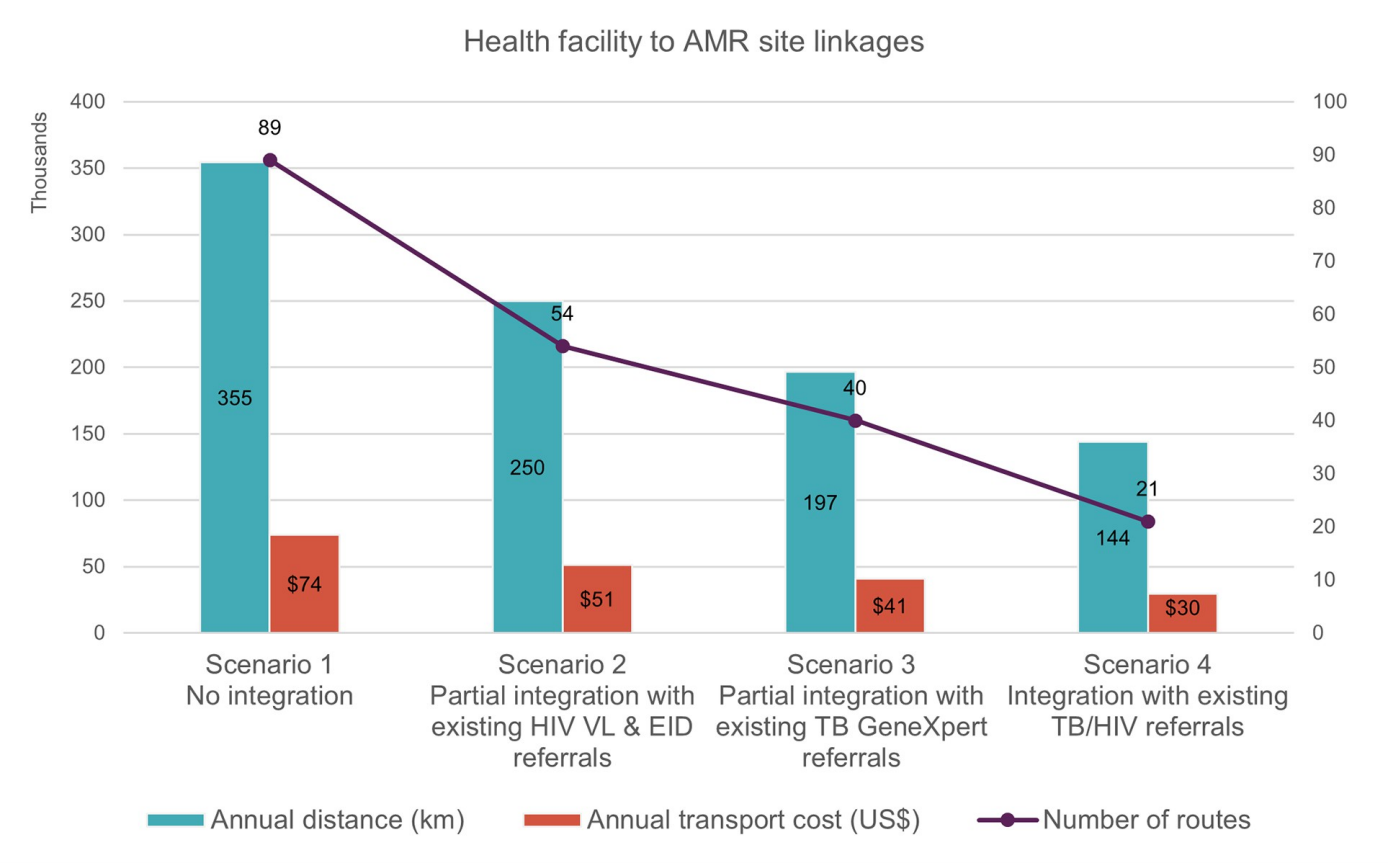
Annual distance, transport cost and number of routes required across optimized scenarios for the first tier of network in Kenya (sample referrals from health facilities to AMR sites). AMR, antimicrobial resistance; EID, early infant diagnosis of HIV; TB, tuberculosis; VL, viral load.

Fig 2 and Table 3 show a comparison of the four optimized scenarios recommended by the DNO analysis for the first tier of the network (referral of patient samples from health facilities to the AMR site), in terms of annual distance, transport costs and number of routes required. The integration of AMR samples with the TB/HIV sample referral systems (scenario 4) was found to optimize the efficiency of the network. Annually, the integration of AMR with the existing TB/HIV sample referral system would save an average of 210,726 km travelled and US$ 44,481. Consequently, when possible, the DNO analysis strongly recommended that referrals with pre-existing sample referral systems are integrated to ensure sustainability and cost savings.

**Table 3:** Distance and cost savings across optimized scenarios for the first tier of network in Kenya (sample referrals from health facilities to AMR sites).

First tier of network: Health facility to AMR site linkages	Scenario 1	Scenario 2	Scenario 3	Scenario 4
	No integration	Partial integration with existing HIV VL & EID referrals	Partial integration with existing TB GeneXpert referrals	Integration with existing TB/HIV referrals
Percentage of integrated routes	0%	39%	55%	76%
Annual distance saved (km)	0	104,716	157,677	210,726
Percentage of annual distance saved	0%	30%	44%	59%
Transport cost (US$)	$73,987	$51,279	$41,177	$29,507
Annual transport cost saved (US$)	0	$22,708	$32,810	$44,481
Percentage of annual transport cost saved	0%	31%	44%	60%

AMR, antimicrobial resistance; EID, early infant diagnosis of HIV; TB, tuberculosis; VL, viral load.

**Fig 3:**
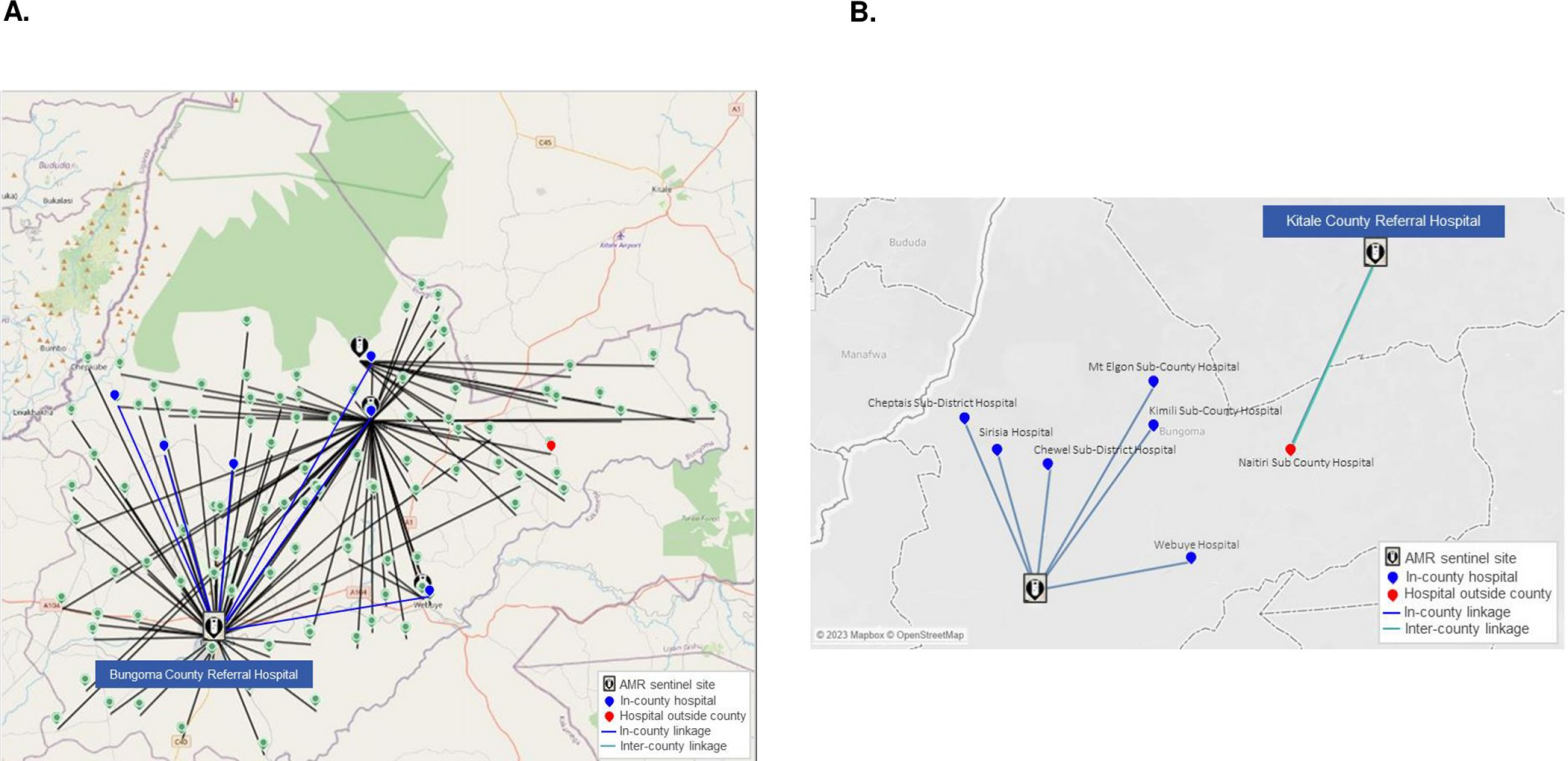
Recommended bacteriology sample referral system in Bungoma County.

An example of the recommended sample referral system for the first tier of the network (sample movements between the hospitals and AMR sentinel sites) is shown in Fig 3 and S1 Table. In the optimized scenario for Bungoma County, Naitiri Sub-County Hospital is linked to the nearest AMR site, which is Kitale County Referral Hospital in Trans Nzoia County. The optimized scenario creates opportunities for transport cost savings through using existing TB and HIV referrals.

Base map and data from OpenStreetMap and OpenStreetMap Foundation.

**Fig 4:**
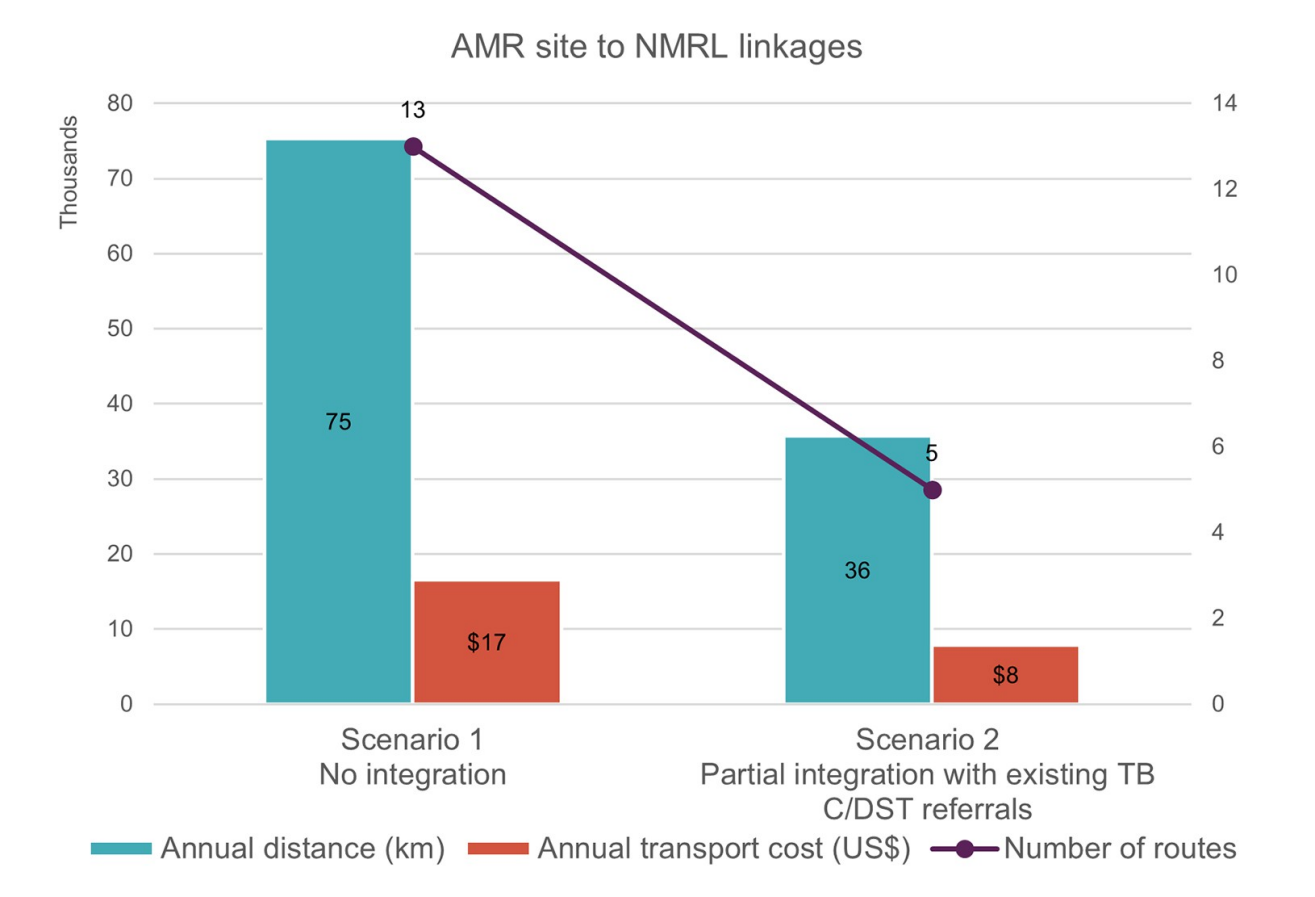
Annual distance, transport cost and number of routes required across optimized scenarios for the second tier of network in Kenya (AMR isolate referrals to the National Microbiology Reference Laboratory). AMR, antimicrobial resistance; C/DST, culture/drug susceptibility testing; NMRL, National Microbiology Reference Laboratory; TB, tuberculosis.

Fig 4 and Table 4 show a comparison of the optimized scenarios recommended by the DNO analysis for isolate transportation to NMRL in terms of annual distance, transport costs and number of routes required. Findings from the analysis indicated that the optimal sample referral system for the second tier of the network–isolate transportation to NMRL from ARM sentinel sites–would be through using the current referral system for TB culture and drug susceptibility testing, which is already in place for 8 AMR sites (S3 Fig). Assuming a transport frequency of twice a month, this scenario (scenario 2 in Fig 4 and Table 5) would result in annual transport cost savings of US$ 8711.

**Table 4:** Distance and cost savings across optimized scenarios for the second tier of network in Kenya (AMR isolate referrals to the National Microbiology Reference Laboratory).

Second tier of network: AMR site to NMRL linkages	Scenario 1	Scenario 2
	No integration	Partial integration with existing TB C/DST referrals
Percentage of integrated routes	0%	62%
Annual distance saved (km)	0	39,596
Percentage of annual distance saved	0	53%
Transport cost (US$)	$16,580	$7869
Annual transport cost saved (US$)	0	$8711
Percentage of annual transport cost saved	0%	53%

AMR, antimicrobial resistance; C/DST, culture/drug susceptibility testing; NMRL, National Microbiology Reference Laboratory; TB, tuberculosis.

**Table 5:** Estimated annual transport cost for the network from hub to regional labs and labs to Central Veterinary Laboratory.

	Scenario 1	Scenario 2	Scenario 3	Scenario 4	Scenario 5	Scenario 6
**Network linkages**	Via hubs	Via hubs	Via hubs	Via hubs	Via hubs	Via hubs
**Sample transport frequency hub-lab**	6 times/week	6 times/week	4 times/ week	4 times/week	3 times/week	3 times/week
**Isolate transport frequency lab—Central Veterinary Laboratory**	Twice/month	Monthly	Twice/month	Monthly	Twice/month	Monthly
**Transport cost (US$) Transport cost with maximum allowable distance constraint (US$)**	**$156,531.46**	**$156,338.65**	**$104,482.85**	**$104,290.04**	**$78,458.55**	**$78,265.73**
	**$120,871.38**	**$120,678.56**	**$80,709.46**	**$80,516.64**	**$60,628.51**	**$60,435.69**

A costing analysis was performed to evaluate the cost of implementing the recommended sample referral systems, using data that were provided by the county authorities and assumptions defined with NASIC on a number of elements (including transport modes, distance travelled, transport frequency, referral routes). When estimating the cost of moving samples from point A to B and excluding other cost elements such as human resources, packaging, supplies and documentation, the estimated annual transport cost was US$ 73,987 for the first tier of the network and US$ 16,580 for the second tier (S2 Table).

### Nepal: Animal health

#### Baseline

Between January and December 2021, a total of 3549 bacteriology and antimicrobial susceptibility tests on faecal, caecal and tissue samples from poultry species in five laboratories were conducted in Nepal; 42% of these were culture tests, 25% were bacterial identification tests and 33% were antimicrobial susceptibility tests. A large proportion of bacteriology testing for animal health in Nepal came from one site, the Central Veterinary Laboratory, which conducted 43% of overall bacteriology testing in the poultry population. Two sites–the Central Veterinary Laboratory and National Avian Disease Investigation Laboratory–were found to undertake almost 70% of the overall testing volume for poultry species in Nepal. However, the Central Veterinary Laboratory was found to receive few isolates (around 10 on an annual basis) from veterinary laboratories each year for confirmatory testing due to a lack of mandatory mechanisms for referral, resources, and staff training.

#### Future state scenarios & recommendations

**Fig 5:**
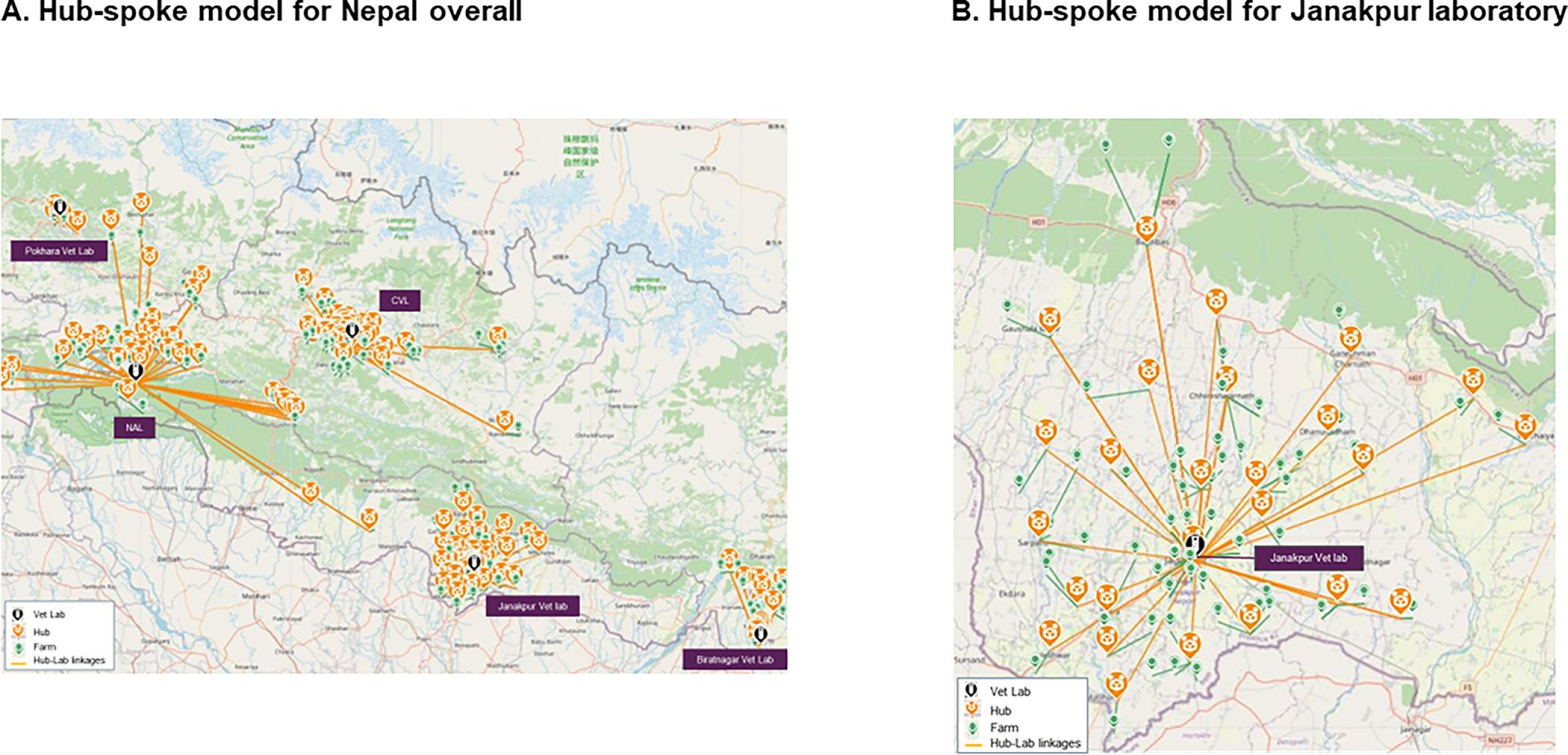
Recommended sample referral system for animal health in Nepal. CVL, Central Veterinary Laboratory; NAL, National Avian Disease Investigation Laboratory.

As Nepal is embarking on active surveillance for the poultry population, the creation of a hub-spoke model for animal health in Nepal was recommended for connecting farms and slaughterhouses to regional veterinary laboratories to limit the distance travelled by farmers. In the model, hubs (samples collection points) are located at local municipality government offices to leverage the presence of veterinary technicians who could be responsible for collecting the samples and transporting them to the testing laboratory. Examples of the recommended hub and spoke sample referral system for the entire network and for Janakpur laboratory are shown in Fig 5.

Base map and data from OpenStreetMap and OpenStreetMap Foundation.

The recommended isolate referral system for the second tier of the network is shown in S4 Fig. With only four regional veterinary laboratories in the model and a low estimated number of isolates (n = 72) to be sent to the Central Veterinary Laboratory on an annual basis, the recommended isolate referral system could ship specimens once or twice a month using a mix of motorbike and airplane as modes of transportation.

A costing analysis was conducted to determine the estimated annual cost associated with the transport of specimens across six scenarios, varying in terms of transport frequency (Table 5). A network model with a maximum allowable distance of 20 km between the farm and hub was also explored. In this model, 63 hubs were necessary instead of the 81 created in the network model where no maximum allowable distance constraint was considered. As there are fewer hubs in the network, the costs are also lower, as seen in Table 5.

## Discussion

In this intervention, the application of DNO created a wealth of insights into the bacteriology and AMR surveillance networks in Kenya and Nepal, and enabled the design of efficient and cost-effective diagnostic networks adapted to each country’s settings and priorities. Specifically, the analysis highlighted key gaps and opportunities to inform the design of specimen referrals in both countries. Findings from the DNO analysis also provided evidence for establishing optimized sample and isolate referral networks and findings will be used to implement a pilot of recommended scenarios before wider scale-up. Furthermore, the analyses highlighted the potential for leveraging existing TB/HIV networks for bacteriology sample and isolate referrals in Kenya for human health.

While DNO has been successfully applied to optimize diagnostic networks for other diseases like TB and HIV [18, 20, 21], this was the first large-scale application of DNO to AMR. The critically high burden of AMR in low- and middle-income countries carries dire consequences for the health of people and animals in such settings, as drug-resistant organisms spread throughout the environment and between countries. Strengthening diagnostic networks and surveillance systems is key to tackling growing rates of AMR [3, 5], and as such, DNO offers lower-resource countries an evidence-based, open-access approach to optimize their diagnostic networks. As scaling up surveillance laboratories is a timely and costly endeavour, DNO can help design and optimize specimen/isolate referral systems to increase access to testing and improve surveillance in the shorter term in resource-limited settings.

Findings from the analyses are being used to guide decision-makers in Kenya and Nepal in budgeting and action planning for the implementation of the recommended referral systems. In Kenya, the national sample referral system technical working group is currently engaging an external provider to provide sample referral services for all specimens, and the DNO outputs will be used to support the design and implementation of the integrated model. In Nepal, the recommended referral network design and proposed route maps will inform a stepwise implementation of a sustained samples and isolate referral network for animal health.

The DNO analyses in Kenya and Nepal also provided a number of learnings and considerations that can be used to guide future applications of DNO to AMR. Firstly, when working on AMR laboratory networks, it is important to consider that AMR programmes are often relatively new in low- and middle-income countries compared with other more established national disease programmes like TB and HIV. In this intervention, DNO was a new concept to the national AMR programmes, which meant the benefits of the approach and specific outputs that countries could use had to be communicated clearly to stakeholders who were unfamiliar with the process.

Early engagement with the country stakeholders was therefore important to align partners on the objectives of and benefits from DNO upfront, and drive engagement with the project. Work was undertaken to highlight the benefits of DNO for the AMR programmes and define scopes for the analyses that took the local context and national priorities into account, to make sure outputs were impactful and useful. Early engagement was also important to identify and address any capacity building needs that stakeholders may have had around conducting a DNO analysis. Building the capacity of the national AMR programmes and key stakeholders around DNO is important to enable an inclusive and country-owned process and ensure that implementable outputs are prioritized during the process. For settings where geospatial analysis is a new concept, an initial laboratory mapping exercise may also be beneficial to secure interest in DNO and give stakeholders the skills to evaluate network designs and refine questions to be addressed by the full DNO analysis.

This intervention also highlights that newer programmes like AMR should utilize data-driven strategic planning to design diagnostic networks as early as possible, to reduce network inefficiencies, avoidable costs, and challenges with access and coverage from the start. Utilizing DNO enables the deployment of diagnostic networks based on need for services rather than geographic or administrative considerations.

Another key consideration when conducting any DNO analysis is that the availability and quality of data significantly affect the resources needed and timelines for conducting a DNO, as well as outcomes of the analysis. Therefore, before undertaking any analysis, it is essential to ascertain that key data inputs, such as geocoordinates of health facilities and testing sites, are available. In terms of AMR specifically, the nature of bacteriology and AMR networks may also present some challenges compared with other disease-specific diagnostic networks such as TB/HIV. In particular, defining tests, testing capacity, device-test combinations and estimating test costs can be challenging, as bacteriology testing comprises a sequence of different steps than cannot be separated from each other. In addition, an important component of bacteriology and antimicrobial susceptibility testing is manual testing (e.g. culture), which is more difficult to model than machine-based testing. Furthermore, as DNO is most effective when embedded in planning/funding cycles, a DNO analysis needs to be conducted at an appropriate time, when insights from the analysis can contribute to the development of evidence-based national strategic plans, funding requests, resource allocation, or procurement and operational planning.

Finally, it is important to note that the value and impact of a DNO analysis will ultimately depend on whether the suggested interventions are adopted and how well they are implemented and monitored. Consequently, plans should be in place to support the practical implementation of interventions and for the monitoring and evaluation of interventions once established. Application of DNO to AMR also presents opportunities to address other potential questions and topics that may be of interest in the future, including sequencing capacity placement, how best to support AMR surveillance efforts, and where diagnostic network capacity for bacteriology and antimicrobial susceptibility testing could be integrated with testing for other diseases to increase access and efficiency.

Limitations of this intervention largely related to the availability of data for the DNO. In Nepal, the analysis was limited in geographic scope as much of the data need for a countrywide analysis (e.g. geocoordinates, laboratory data) were not available. Some of the approaches utilized to overcome data issues included using approximate farm locations, estimating annual data for 2021 using quarterly/biannual data, and modelling testing capacity. These factors could have affected the precision of the recommended network designs in Nepal, but provided stakeholders with an initial analysis that would have not been available otherwise.

Overall, the project highlights the benefits of using a data-driven approach for designing efficient diagnostic networks, to ensure better health resource allocation while maximizing the impact and equity of health interventions. Given the critical need to strengthen AMR laboratory and surveillance capacity, network mapping and optimization should be considered an integral part of diagnostic strategic planning in the future.

## Supporting information

S1 Checklist Inclusivity in global research. (DOCX)

S1 Table Recommended bacteriology sample referral system for hospitals in Bungoma County. (DOCX)

S2 Table Distance and cost savings across optimized scenarios for the second tier of network in Kenya (AMR isolate referrals to the National Microbiology Reference Laboratory). (DOCX)

S1 Fig Isolate referrals at baseline in Kenya. (TIF)

S2 Fig Baseline sample referral network for human health in Kenya. (TIF)

S3 Fig Optimized isolate referral system in Kenya recommended by model. (TIF)

S4 Fig Recommended animal health sample referral system for isolates in Nepal. (TIF)
