# Supplementary material for: Application of diagnostic network optimization in Kenya and Nepal to design integrated, sustainable and efficient bacteriology and antimicrobial resistance surveillance networks
Source: PLOS Glob Public Health. 2023 Dec 6;3(12):e0002247. doi: 10.1371/journal.pgph.0002247 (PMC10699636; doi:10.1371/journal.pgph.0002247)
Supplement: S1 Table — (DOCX) [file pgph.0002247.s002.docx]

| **#** | **Hospitals** | **County** | **Distance** | **Transport mode** | **Frequency** | **Cost (US$)** |
| --- | --- | --- | --- | --- | --- | --- |
| 1 | **Cheptais Sub-District Hospital** | Bungoma | 38.5 km | Boda boda* | Three times per week | $1321 |
| 2 | **Sirisia Hospital** | Bungoma | 29 km | Boda boda | Three times per week | $990 |
| 3 | **Chewel Sub-District Hospital** | Bungoma | 24.9 km | Boda boda | Three times per week | $855 |
| 4 | **Mt Elgon Sub-County Hospital** | Bungoma | 48 km | Boda boda | Five times per week | $1647 |
| 5 | **Kimili Sub-County Hospital** | Bungoma | 41 km | Boda boda | Three times per week | $2341 |
| 6 | **Webuye Hospital** | Bungoma | 32.4 km | Boda boda | Five times per week | $1856 |

*Local motorbike taxis
