# Supplementary material for: Application of diagnostic network optimization in Kenya and Nepal to design integrated, sustainable and efficient bacteriology and antimicrobial resistance surveillance networks
Source: PLOS Glob Public Health. 2023 Dec 6;3(12):e0002247. doi: 10.1371/journal.pgph.0002247 (PMC10699636; doi:10.1371/journal.pgph.0002247)
Supplement: S2 Table — (DOCX) [file pgph.0002247.s003.docx]

| **AMR site** | **County** | **Distance AMR site-hub (km)** | **Transport mode AMR site-hub** | **Distance hub- NMRL (km)** | **Transport mode hub-NMRL** | **Frequency** | **Cost (US$)** |
| --- | --- | --- | --- | --- | --- | --- | --- |
| Bungoma County Referral Hospital | Bungoma | 0.8 | Boda boda | 421 | G4S | Twice/ month | $2227 |
| Coast General Teaching and Referral Hospital | Mombasa | 5.7 | Boda boda | 488 | G4S | Twice/ month | $2607 |
| JOOTRH | Kisumu | 4.2 | Boda boda | 349 | G4S | Twice/ month | $1865 |
| Kenyatta National Hospital | Nairobi | 0 | NA | 2 | G4S | Twice/ month | $11 |
| Kitale County Referral Hospital | Trans Nzoia | 1.2 | Boda boda | 391 | G4S | Twice/ month | $2071 |
| Machakos Level 5 hospital | Machakos | 1.9 | Boda boda | 64 | G4S | Twice/ month | $348 |
| Makueni County Referral Hospital | Makueni | 0.4 | Boda boda | 134 | G4S | Twice/ month | $710 |
| Malindi Sub-County Hospital | Kilifi | 2.8 | Boda boda | 498 | G4S | Twice/ month | $2644 |
| Moi Teaching and Referral Hospital | Uasin Gishu | 1.2 | Boda boda | 320 | G4S | Twice/ month | $1696 |
| Muranga County Referral Hospital | Muranga | 0.7 | Boda boda | 87,8 | G4S | Twice/ month | $467 |
| Nakuru Provincial General Hospital | Nakuru | 1.9 | Boda boda | 167 | G4S | Twice/ month | $892 |
| Nyeri County Referral Hospital | Nyeri | 0.02 | NA | 148 | G4S | Twice/ month | $782 |
| Thika Level 5 Hospital | Kiambu | 1.6 | Boda boda | 48 | G4S | Twice/ month | $262 |
