# Supplementary figures and images for: Application of diagnostic network optimization in Kenya and Nepal to design integrated, sustainable and efficient bacteriology and antimicrobial resistance surveillance networks
Source: PLOS Glob Public Health. 2023 Dec 6;3(12):e0002247. doi: 10.1371/journal.pgph.0002247 (PMC10699636; doi:10.1371/journal.pgph.0002247)

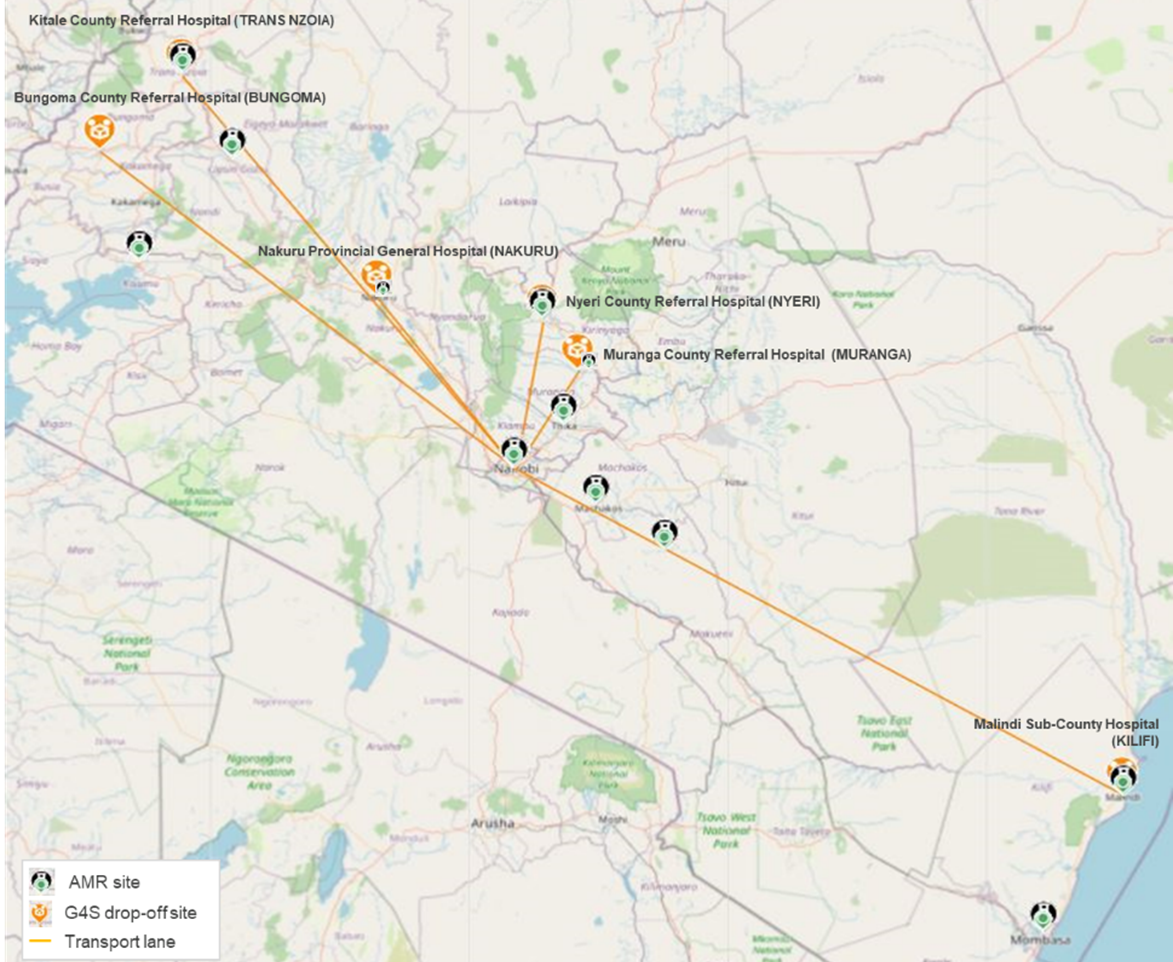

Supplement: S1 Fig — (TIF) [file pgph.0002247.s004.tif]

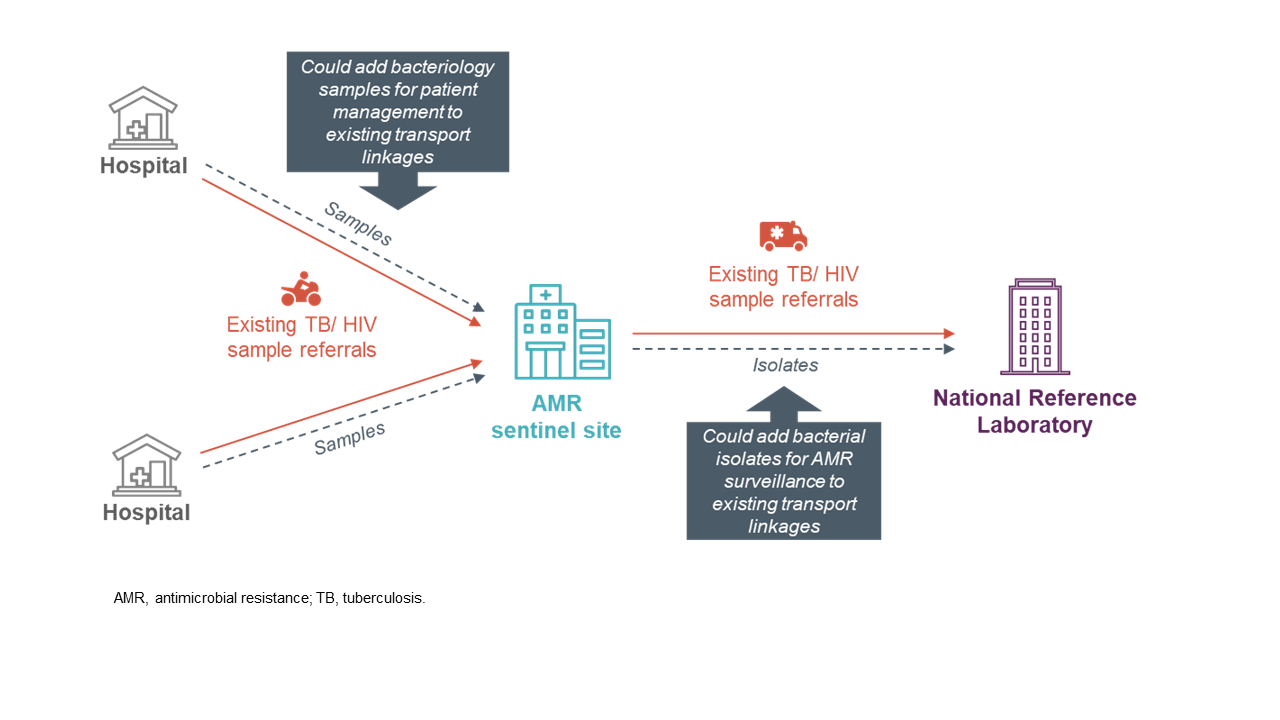

Supplement: S2 Fig — (TIF) [file pgph.0002247.s005.tif]

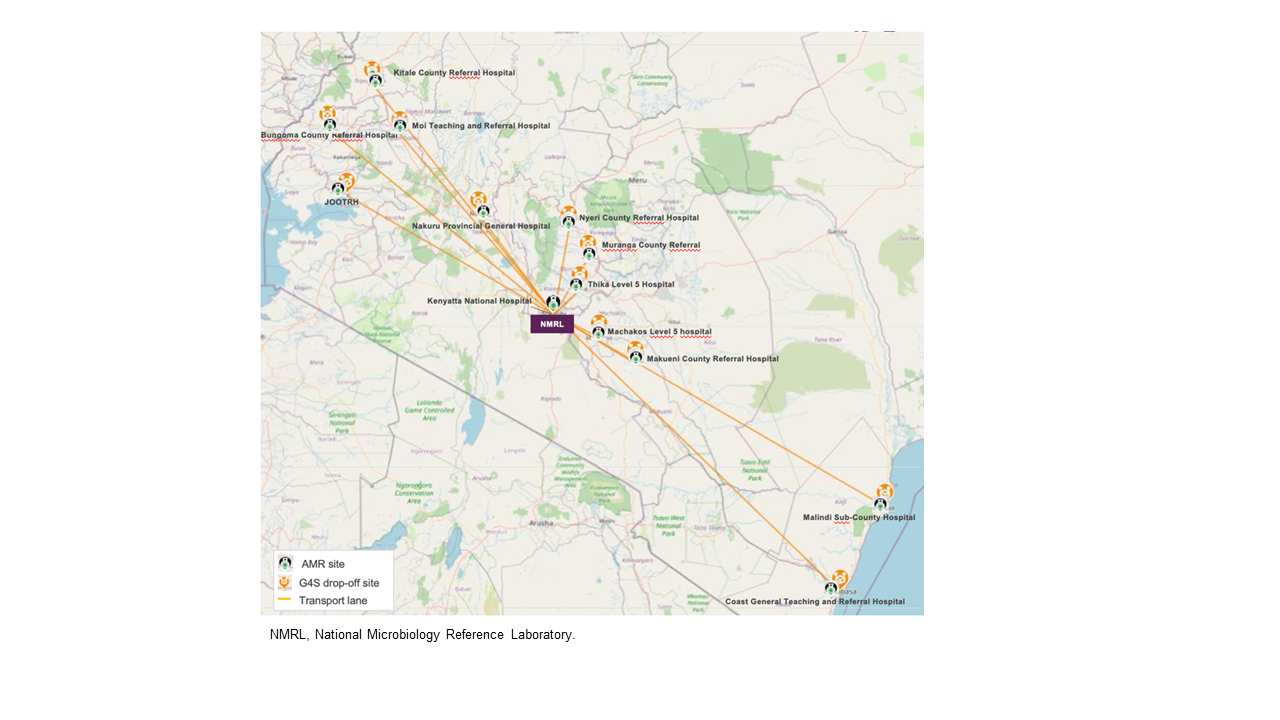

Supplement: S3 Fig — (TIF) [file pgph.0002247.s006.tif]

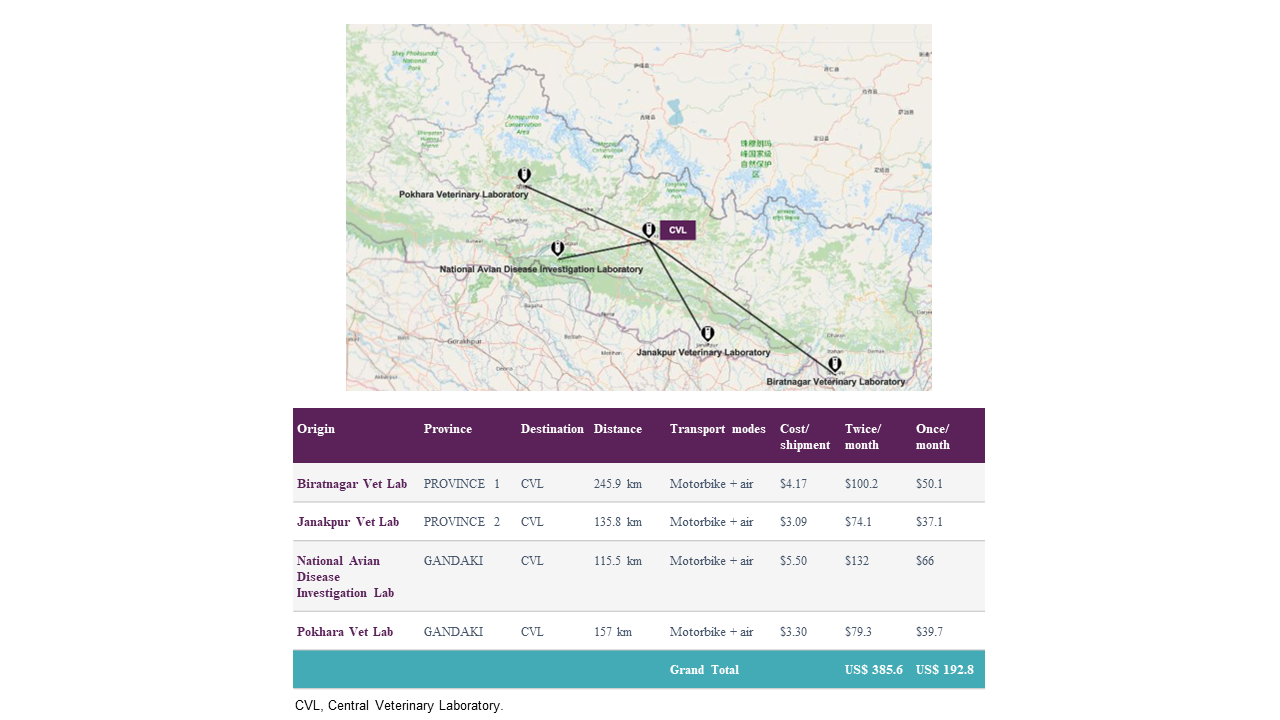

Supplement: S4 Fig — (TIF) [file pgph.0002247.s007.tif]
